# First Wave of COVID-19 in French Patients with Cystic Fibrosis

**DOI:** 10.3390/jcm9113624

**Published:** 2020-11-10

**Authors:** Harriet Corvol, Sandra de Miranda, Lydie Lemonnier, Astrid Kemgang, Martine Reynaud Gaubert, Raphael Chiron, Marie-Laure Dalphin, Isabelle Durieu, Jean-Christophe Dubus, Véronique Houdouin, Anne Prevotat, Sophie Ramel, Marine Revillion, Laurence Weiss, Loic Guillot, Pierre-Yves Boelle, Pierre-Régis Burgel, on behalf of the French Cystic Fibrosis Reference Network Study Group

**Affiliations:** 1 Pediatric Pulmonology Department and Pediatric CF Center, Assistance Publique Hôpitaux de Paris (APHP) Hôpital Trousseau, 75012 Paris, France; 2 Centre de Recherche Saint‑Antoine (CRSA), INSERM UMR_S938, Sorbonne Université, 75012 Paris, France; astrid-donald.kemgang-fankem@inserm.fr (A.K.); loic.guillot@inserm.fr (L.G.); 3 Pulmonology Department and CF Center, Hôpital Foch, 92151 Suresnes, France; s.demiranda@hopital-foch.com; 4 Association Vaincre la Mucoviscidose, 75013 Paris, France; llemonnier@vaincrelamuco.org; 5 Pulmonology Department and CF Adult Center, Hôpital Nord, Assistance Publique Hôpitaux de Marseille (APHM), 13915 Marseille, France; MartineLouise.REYNAUD@ap-hm.fr; 6 Aix-Marseille Université, Institut de Recherche pour le Développement (IRD), IHU Méditerranée Infection, MEPHI, 13005 Marseille, France; 7 CF Center, Hôpital Arnaud de Villeneuve, CHU de Montpellier, 34295 Montpellier, France; r-chiron@chu-montpellier.fr; 8 Pediatric CF Center, Hôpital Jean-Minjoz, CHU de Besançon, 25030 Besançon, France; mldalphin@chu-besancon.fr; 9 Internal Medicine Department and Adult CF Center, Hospices Civils de Lyon, EA 7425 HESPER, Université de Lyon, 69495 Lyon, France; isabelle.durieu@chu-lyon.fr; 10 Pediatric Pulmonology Department and Pediatric CF Center, APHM, 13385 Marseille, France; JeanChristophe.DUBUS@ap-hm.fr; 11 Pediatric CF Center, APHP Hôpital Robert Debré, 75019 Paris, France; veronique.houdouin@aphp.fr; 12 Adult CF Center, Hôpital Calmette and University Lille, 59037 Lille, France; anne.prevotat@chru-lille.fr; 13 Pediatric and Adult CF Center, 29680 Roscoff, France; sophie.ramel@ildys.org; 14 Pediatric CF Center, Hôpital Jeanne de Flandres, CHU Lille, 59037 Lille, France; mrevillion@ch-lens.fr; 15 Pediatric CF Center, Hôpitaux Universitaires de Strasbourg, 67098 Strasbourg, France; Laurence.WEISS@chru-strasbourg.fr; 16 Institut Pierre Louis d’Epidémiologie et de Santé Publique, INSERM, APHP, Sorbonne Université, 75012 Paris, France; pierre-yves.boelle@upmc.fr; 17 Respiratory Medicine and National Reference CF Center, AP-HP Hôpital Cochin, 75014 Paris, France; pierre-regis.burgel@aphp.fr; 18 Institut Cochin, Inserm U-1016, Université de Paris, 75014 Paris, France

**Keywords:** COVID-19, SARS-CoV-2, Cystic Fibrosis, acute respiratory distress syndrome, lung transplant

## Abstract

Viral infections are known to lead to serious respiratory complications in cystic fibrosis (CF) patients. Hypothesizing that CF patients were a population at high risk for severe respiratory complications from SARS-CoV-2 infection, we conducted a national study to describe the clinical expression of COVID-19 in French CF patients. This prospective observational study involves all 47 French CF centers caring for approximately 7500 CF patients. Between 1 March and 30 June 2020, 31 patients were diagnosed with COVID-19: 19 had positive SARS-CoV-2 RT-PCR in nasopharyngeal swabs; 1 had negative RT-PCR but typical COVID-19 signs on a CT scan; and 11 had positive SARS-CoV-2 serology. Fifteen were males, median (range) age was 31 (9–60) years, and 12 patients were living with a lung transplant. The majority of the patients had CF-related diabetes (*n* = 19, 61.3%), and a mild lung disease (*n* = 19, 65%, with percent-predicted forced expiratory volume in 1 s (ppFEV_1_) > 70). Three (10%) patients remained asymptomatic. For the 28 (90%) patients who displayed symptoms, most common symptoms at admission were fever (*n* = 22, 78.6%), fatigue (*n* = 14, 50%), and increased cough (*n* = 14, 50%). Nineteen were hospitalized (including 11 out of the 12 post-lung transplant patients), seven required oxygen therapy, and four (3 post-lung transplant patients) were admitted to an Intensive Care Unit (ICU). Ten developed complications (including acute respiratory distress syndrome in two post-lung transplant patients), but all recovered and were discharged home without noticeable short-term sequelae. Overall, French CF patients were rarely diagnosed with COVID-19. Further research should establish whether they were not infected or remained asymptomatic upon infection. In diagnosed cases, the short-term evolution was favorable with rare acute respiratory distress syndrome and no death. Post-lung transplant patients had more severe outcomes and should be monitored more closely.

## 1. Introduction

Cystic fibrosis (CF) is the most common severe autosomal recessive genetic disease in Caucasians, affecting around 7500 patients in France [1]. CF is caused by variants in the gene encoding the cystic fibrosis transmembrane conductance regulator (CFTR) [2]. CFTR is an epithelial cell anion channel that plays a major role in the transport of chloride, as well as other anions including bicarbonates (HCO_3_^−^), thiocyanate (SCN^−^), iodide (I^−^), and glutathione. The disease affects many organs, such as the pancreas, liver, intestine, and, most critically, the lungs [3,4]. Lung disease remains the major cause of morbidity and mortality in CF, with a progressive decline of lung function due to a vicious cycle of airway infections and inflammation [5,6]. Among the pathogens infecting the airways in CF patients, viruses are known to contribute to the deterioration of respiratory function, and patients with CF are among those at risk of serious respiratory complications from infection with influenza A virus [7].

Since December 2019, the coronavirus disease 2019 (COVID-19) outbreak has drastically changed the sanitary face of the world. As of 27 October 2020, more than 40 million people have been infected with the new severe acute respiratory syndrome coronavirus 2 (SARS-CoV-2). Recent seroprevalence studies show that by mid-May 2020, 5% of the French population had been infected with the virus [8].

The impact of SARS-CoV-2 infection in patients with CF was unknown. Given the serious respiratory complications caused by viral infections, CF patients were expected to be at higher risk of severity. We therefore started a prospective observational study involving all 47 CF French centers in March 2020. The main objective was to describe the clinical expression of COVID-19 in French patients with CF.

## 2. Materials and Methods

Following the spread of COVID-19 outbreak in France, all 47 French CF centers, which follow approximately 7500 patients, agreed to conduct a prospective observational study (MR004-2218155) to describe the clinical manifestations of SARS-CoV-2 infection in people with CF [1]. The study was approved by the Institutional Review Board of the French Society for Respiratory Medicine (Société de Pneumologie de Langue Française, # CEPRO_2020-013). All patients received information about the study, but in accordance with French laws for observational studies, informed consent was not required.

At the time of the study, SARS-Cov-2 testing in France was reserved for the most-at-risk people: symptomatic health professionals, symptomatic elderly people, and people with severe respiratory difficulties or comorbidities, i.e., chronic diseases such as CF, hospitalized people, and any new cluster. Testing consisted initially of performing SARS-CoV-2 RT-PCR in nasopharyngeal swabs, with serological tests only becoming available in May 2020.

Data of CF patients infected by SARS-CoV-2 were collected in a dedicated CF-COVID registry, which is nested within the French CF registry (maintained by the French CF patient association, Vaincre la Mucoviscidose). To account for the potential lack of performance of the real-time reverse transcription (RT)-PCR for SARS-CoV-2 due to sampling quality or kinetics (sensitivity < 60%) [9,10,11], a body of evidence was used for assessing COVID-19 diagnosis. Criteria for a positive case included: (i) positive SARS-CoV-2 RT-PCR in nasopharyngeal swabs; and/or (ii) typical aspects of COVID-19 on thoracic high-resolution computed tomography (HRCT), and/or (iii) positive serology. Typical HRCT aspects include ground-glass opacities (GGO), consolidation, bilateral and peripheral distribution of the lesions, as well as round aspect of the lesions [12,13]. Additionally, a standardized report was proposed by the French Radiology Society (https://ebulletin.radiologie.fr/comptes-rendus-covid-19) to homogenize HRCT reports of patients with COVID-19.

COVID-19 transmission history, clinical, biological, radiological information, as well as medical evolution were collected in the registry. Continuous data were expressed as median and range values, while categorical data were expressed as numbers and proportions (%). Descriptive statistics were used for all study variables. We used the Fisher’s exact test for the comparison of categorical and qualitative data, and the Mann Whitney’s non-parametric test for continuous variables. The methods used to compute and compare the expected number and age of SARS-Cov-2 infected CF cases to the observed ones are described in the Supplementary Materials. We used data from the French CF registry [1] to compare the clinical characteristics of the SARS-CoV-2 infected CF patients to the overall French CF population. A P-value of less than 5% was interpreted as evidence of a statistically significant difference. The analyses were carried out using the software R (http://www.R-project.org/).

## 3. Results

Between 1 March and 30 June 2020, 31 patients were diagnosed with COVID-19: 19 had positive SARS-CoV-2 RT-PCR in nasopharyngeal swabs; 1 had negative RT-PCR but typical COVID-19 aspects on a CT scan; and 11 had positive SARS-CoV-2 serology. Among the patients with a positive serology, 3 were asymptomatic (see Table S2 for details).

### 3.1. Evaluation of the Expected Number and Age of SARS-Cov-2 Infected CF Patients

We compared the observed number and age of COVID-19 CF cases to the expected ones (see Supplementary Materials). Had the risk of SARS-Cov-2 infection been the same in the CF population as that in the general population, approximately 470 SARS-CoV-2 infections should have occurred before 30 June 2020. Given that only 31 cases were reported, the overall risk reduction with respect to the general population was 93% (Table S1). The age distribution of CF cases suggests that fewer young cases were observed than expected (Figure S1).

### 3.2. Clinical Characteristics at COVID-19 Onset

We compared baseline clinical characteristics of the 31 SARS-CoV-2 infected CF patients to the overall French CF population from the national registry [1]. This registry reports data of the CF patients who had at least one visit in a CF center in 2017 (*n* = 6913, >90% of all French CF cases). As shown in Table S3, CF patients with COVID-19 CF were older than the overall CF population. There were differences in comorbidities consistent with older age, including more frequent *Pseudomonas aeruginosa* chronic colonization, CF-related diabetes, and the use of treatments such as oral corticosteroids and azithromycin.

**Figure 1:**
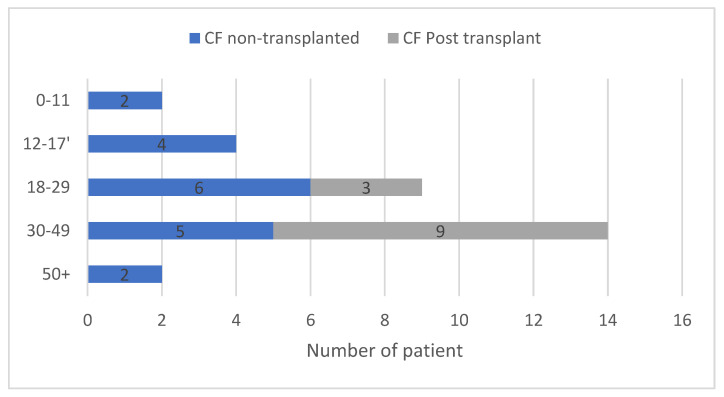
Distribution of the patients with cystic fibrosis at SARS-CoV-2 infection onset according to age-categories. Among the 31 included patients, a majority were adults (≥18 years) with 14 patients (45%) aged between 30–49 years-old, and 9 (29%) between 18–29 years-old. Only 6 patients (19%) were children.

The clinical characteristics of the 31 CF patients at SARS-CoV-2 infection onset are described in Table 1. Fifteen were males, the median age was 31 years old (range: 9–60), and 12 were post-lung transplant patients (older than the non-transplanted ones, *p* = 0.03). Figure 1 depicts the number of patients according to age category, showing a majority of adults, with only six CF children (under 18 years old) diagnosed with SARS-CoV-2 infection.

**Figure 2:**
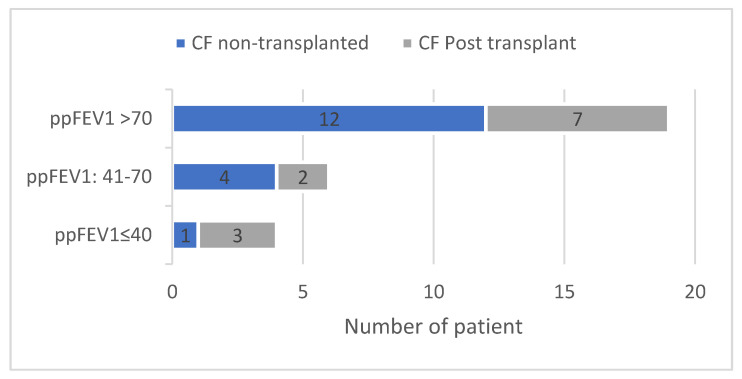
Distribution of baseline lung function in 31 patients with CF at SARS-CoV-2 infection onset. At SARS-CoV-2 infection onset, among the 31 patients, the majority had preserved lung function, 19 patients (65%) having a percent-predicted forced expiratory volume in one second (ppFEV_1_) that was greater than 70.

The patients were mostly exocrine pancreatic insufficient (*n* = 28, 90%), and the majority had a mild lung disease (*n* = 19, 65% with a percent-predicted forced expiratory volume in 1 s (ppFEV_1_) greater than 70) (Figure 2). The CF comorbidities are described in Table 1: 42% were chronically infected by *P. aeruginosa*; and 61.3% had CF-related diabetes. Overall, only six (19.4%) had systemic arterial hypertension; but five out of these six were post-lung transplant patients (*p* = 0.02). At infection onset, 16 patients (51%) were under oral corticosteroids, including all the 12 post-lung transplant patients, as oral corticosteroids were part of their immunosuppressive drug regimen (comparison non-transplanted vs. post-transplanted patients *p* < 0.0001). Eighteen (58%) patients overall were under long-term azithromycin at low dose, and only one post-lung transplant patient took nonsteroidal anti-inflammatory drugs (NSAIDs).

**Figure 3:**
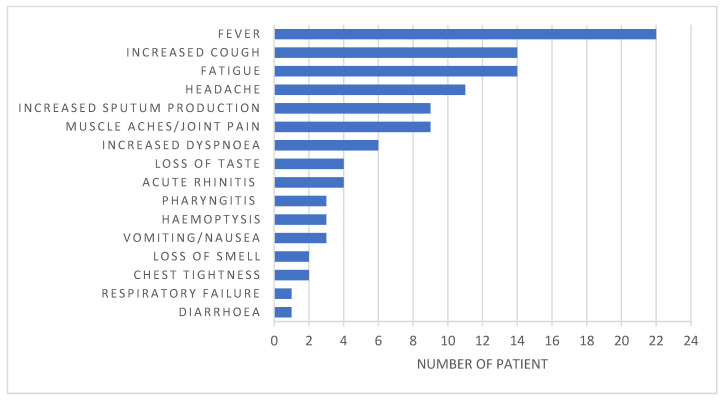
Distribution of the main symptoms at SARS-CoV-2 infection at onset in the patients with cystic fibrosis. Among the 31 patients, 28 (90%) had symptoms of SARS-CoV-2 infection at onset, whereas 3 (10%) remained asymptomatic.

The clinical symptoms at COVID-19 onset are described in Figure 3. Among the included 31 patients, 3 (10%) remained asymptomatic. For the 28 (90%) patients who displayed symptoms, most common at onset were fever (*n* = 22, 78.6%), fatigue (*n* = 14, 50%), and increased cough (*n* = 14, 50%). Interestingly, dyspnea was present in only 6 (21%) patients, whereas gastro-intestinal symptoms were found in 3 (11%) patients.

### 3.3. Evolution and Treatments of COVID-19

Outcomes and treatments of COVID-19 are described in Table 2. Nineteen patients (61%) required being hospitalized, including 11 out of the 12 post-lung transplant patients (comparison non-transplanted vs. post-lung transplant patients: *p* = 0.008). The median duration of hospitalization was 10 days overall (range: 2–41). Seven (22%) patients required additional oxygen therapy, including six post-lung transplant patients (*p* = 0.006), and four (16%) patients required hospitalization in an Intensive Care Unit (ICU). Three out of the four patients who required ICU care were post-lung transplant patients, the fourth being a 9-year-old child with a mild lung disease pre-COVID and a favorable evolution (see Tables S4 and S5 for details).

Ten (32%) patients developed complications, including four with respiratory exacerbations. Two post-lung transplant patients developed acute respiratory distress syndrome (ARDS) and required ICU care. One of them was treated with high-flow nasal canula oxygen therapy for 11 days, systemic corticosteroids, and anti-interleukin (IL)-6 (Sarilumab). The second one required invasive ventilation for 9 days. No patient received antiviral treatments, and only two post-lung transplant patients received hydroxychloroquine (Table 2). All the patients recovered and were discharged home without noticeable short-term sequelae.

## 4. Discussion

In this national multicenter study, we describe the clinical expression of COVID-19 in French CF patients over the first few months of the pandemic. Given the serious respiratory complications caused by viral infections in CF patients, patients were expected to be at higher risk of COVID-19 and higher risk of severity [7]. However, the number of COVID-19 cases in CF patients was rather low, and severe disease occurred only in a limited number of patients, especially in post-lung transplant patients. Similarly to the general population, children with CF were rarely infected [14,15]. These data suggest that the risk of contracting COVID-19 is not increased in patients with CF, and that the course of COVID-19 does not differ in people with CF as compared to that of the general population. However, post-lung transplant patients need to be closely monitored as their evolution tends to be more severe.

### 4.1. The Incidence of COVID-19 is not Increased in CF Patients

In the present study, we report 31 cases of COVID-19 among the 7500 French CF patients, corresponding to a cumulated incidence of 0.41% by July 2020. The risk of infection was reduced by up to 93% with respect to the general population in the same period. Moreover, we found fewer young CF infected cases than expected. These data will have to be confirmed by seroprevalence studies. For the present, they agree with previous reports that suggested that CF subjects were not at increased risk of developing COVID-19 compared to the general population [16,17]. This may be only due to the fact that CF patients have always paid close attention to infection control and social distancing [18]. Moreover, at the very beginning of the pandemic, CF centers worldwide reorganized their classical care to “keep cystic fibrosis patients out of the hospital” as stated by Dasenbrook [19]. The North-American and European recommendations were as follows: (i) to start CFTR modulators as soon as possible, to reduce pulmonary exacerbations and hospitalizations; (ii) to practice telemedicine as much as possible; (iii) to reinforce adherence to already prescribed regimens; (iv) to promptly and aggressively treat CF exacerbations to prevent avoidable hospitalizations; and (v) to continue to communicate with the patients by emails, phone calls, etc. [18,19]. These measures were applied by the CF centers worldwide and seem to have been beneficial for patients with CF.

The role of azithromycin in the low incidence of COVID-19 in the CF population has been questioned but not confirmed so far. Indeed, many CF patients are treated with low-dose long-term azithromycin, which is known to suppress viral infection and lung inflammation and which inhibits the activity of FURIN, one of the serine proteases involved in the facilitation of SARS-CoV-2 cellular entry, via the ACE2 receptor [20,21]. Moreover, although there are no randomized clinical trials supporting the prescription of azithromycin, it has been widely used (with or without hydroxychloroquine) in COVID-19 treatment because of its immunomodulatory and antiviral properties [22,23,24]. In this study, 18 (58%) patients were under long-term azithromycin treatment before SARS-Cov-2 infection, and 5 (16%) were additionally treated when diagnosed. As such, 23 (74%) patients received azithromycin at some point during the course of COVID-19, and its role in the low incidence, as well as in the benign evolution of COVID-19 in this population, could be further investigated.

### 4.2. COVID-19 Severity Seems not to be Increased in Patients with Cystic Fibrosis in Comparison with other Viral Infections

The course of COVID-19 appeared to be generally mild in French CF patients, with only a minority of patients developing severe disease that required ventilatory support and ICU care. We were surprised to observe that CF patients who developed COVID-19 were less severely impacted compared to patients with other viral respiratory infections, confirming previous data [21]. Additionally, we found that patients with COVID-19 were often not considered to have pulmonary exacerbations, whereas several studies have shown that viral infections cause up to 60% of acute pulmonary exacerbations in patients with CF [7,25,26,27,28,29,30]. Various viruses have been incriminated for this, such as influenza, respiratory syncytial virus (RSV), and rhinovirus [7,25,26,27,28,29,30]. For example, the H1N1-influenza pandemic in 2009 led to significant morbidity in patients with CF, with worsening of lung function decline and decreased survival [25]. A large analysis of data from the US CF Foundation Patient Registry merged with data from the Centers for Disease Control showed a strong correlation between pulmonary exacerbations and RSV infection in children with CF [27]. Rhinovirus-induced pulmonary exacerbations are also common in CF and associated with impaired virus clearance by CF airways [28]. The exact mechanisms of how these respiratory viruses affect lung disease in CF are not fully understood. Various factors have been incriminated, such as a possible reduced antiviral immunity by airway epithelial cells, resulting in increased viral replication [31,32]. Several studies have also underlined that bacterial airways infections, such as *Pseudomonas aeruginosa* infections, are often preceded by viral infections [33]. RSV and *P. aeruginosa* coinfections have been shown to accelerate lung function decline and to increase morbidity and mortality [34]. Long-term studies will be necessary to fully appreciate the impact of COVID-19 on lung function decline in people with CF.

Despite the high severity of many respiratory viral infections in CF compared to non-CF individuals, and the fact that SARS-CoV-2 leads to significant morbidity and mortality, especially in patients with preexisting medical conditions, we were surprised to observe, similarly to others, that it did not cause worse outcomes in our CF cohort [16,17,18,21]. As of 24 September 2020, the European CF registry had collected data from 162 CF patients with COVID-19 all over Europe, among whom 90% had a mild infection severity (https://www.ecfs.eu/covid-cf-project-europe). Only eight patients needed ICU admission, with five cases described as critical, and three patients died. Interestingly, similarly to the international [16] but not to the Spanish study [17], we observed an overrepresentation of post-lung transplant patients (38.7%). The international study highlighted that post-transplant patients might have been more frequently tested because of specific monitoring protocols in their lung transplant centers. Here, we observed that, compared to the non-transplanted patients, the post-lung transplant patients were older, had more frequent systemic arterial hypertension, tended to have a higher frequency of diabetes, and were more frequently under oral corticosteroids treatment (as part of their immunosuppressive drug regimen). We also observed that evolution of COVID-19 appeared more severe in some of the post-lung transplant patients. Indeed, almost all required hospitalization (11 out of the 12 post-lung transplant patients), and among the 4 patients who required ICU care, 3 (75%) were post-lung transplant patients, with 2 developing an ARDS. So far, limited data are available concerning transplant patients and COVID-19 in the general population. It is recognized that, as post-transplant patients receive immunosuppressive therapy, they are at the highest risk of severe illness from COVID-19 [35]. However, as all these reports represent early data from the COVID-19 pandemic, future studies will be necessary to confirm these findings.

### 4.3. Immune and Epithelial Response to SARS-CoV-2 in Cystic Fibrosis

Although mechanistic studies are just beginning, it is established that SARS-CoV-2 infects airway epithelial cells through the ACE2 receptor, facilitated by two specific host serine proteases: TMPRSS2 and FURIN [36,37,38]. These processes might be altered in CF [20]. Indeed, the FURIN enzyme has been shown to regulate ENac, a sodium channel upregulated in CF, where it participates in the thickness of the airway mucus and subsequent lung pathophysiology [39]. Moreover, the CF lung contains high levels of serine protease inhibitors predicted to reduce the ability of TMPRSS2 to facilitate SARS-CoV-2 entry into airway epithelial cells [21]. Altogether, this would suggest that SARS-CoV-2 processing might be influenced by CF-affected cellular processes that may mitigate COVID-19 expression in the CF population.

It is now recognized that innate immune cells are able to modulate their response upon reinfection, a phenomenon called “trained immunity” [40,41]. Moreover, memory cells allow the immune system to provide a quicker and stronger response to viral re-infection, further providing a cross-protection against other viruses. This “trained” innate immune answer can also be enhanced by various infections, independently from the pathogen they specifically target [42]. In comparison to the general population, patients with CF are highly infected by various pathogens, i.e., bacteria, viruses, as well as fungi, which may account for the role of this trained immunity in the puzzling moderate severity of COVID-19 in patients with CF [43].

### 4.4. Strengths and Limitations

The present study was conducted prospectively and was based on direct reporting of cases that occurred in all 47 centers composing the French CF Reference Center Network, covering all pediatric and adult CF patients in France. This network also includes the transplant centers that follow CF patients after lung transplant, insuring representation of both non-transplanted and transplanted CF patients. The standardized study questionnaire was distributed to the centers at the start of the pandemic outbreak in France, and the study was nested within the French CF Registry. We also recognize limitations. First, the pandemic is still developing at the time of writing (September 2020), and our study represents only the situation in the first few months. Second, the study used a definition of cases that required either positive SARS-Cov-2 PCR, a typical CT scan, or a positive serology. Thus, patients who were not tested for SARS-Cov-2 virus or antibodies, or who did not have a CT scan performed were not reported in this study. The three cases who had a positive serology but no symptoms confirmed that COVID-19 may be asymptomatic in CF patients (similarly to the general population) [44]. Although it is likely that the present design led to missing mild cases, this is unlikely to alter our conclusion, as the present series of cases represent the most severe cases that occurred in CF patients in France.

## 5. Conclusions

It remains difficult to estimate the susceptibility of CF patients to SARS-CoV-2 infection, because they are used to masks, hand hygiene, and social distancing to limit exposure and avoid infection. This observational study shows that CF patients were rarely diagnosed with COVID-19. Further studies will establish if they were not infected or were asymptomatic and undiagnosed. We also observed that the short-term evolution was favorable with rare ARDS and no death, but that post-lung transplant patients need to be closely monitored as their evolution tends to be more severe. As the pandemic is far from resolved, close monitoring of CF cohorts will be necessary to fully understand the short-term impact and long-term consequences of COVID-19 in people with CF.

## Figures and Tables

**Table 1:** Clinical characteristics of the 31 patients with cystic fibrosis at SARS-CoV-2 infection onset.

Clinical Characteristics	All Patients	Non-Transplanted Patients	Post-Lung Transplant Patients	Non-Transplanted vs. Post-Lung Transplant Patients
	*n* = 31	*n* = 19	*n* = 12	*p*-Value
Male, *n* (%)	15 (48.4)	8 (42.1)	7 (58.3)	0.47
Age (years), median (range)	31 (9–60)	27 (9–60)	39 (19–48)	0.03
Exocrine pancreatic insufficiency, *n* (%)	28 (90.3)	17 (89.5)	11 (91.7)	1
***CFTR* mutations**				0.88
F508del homozygotes, *n* (%)	10 (32.3)	7 (36.8)	3 (25)	
F508del heterozygotes, *n* (%)	16 (51.6)	9 (47.4)	7 (58.3)	
Other	5 (16.1)	3 (15.8)	2 (16.7)	
Influenza vaccine in the past 12 months, *n* (%)	24 (77.4)	17 (89.5)	7 (58.3)	0.08
ppFEV_1_^1^, median (range)	79 (27–121)	78.5 (37–121)	76 (27–114)	0.68
BMI ^2^, median (range)	20.8 (14.4–26.8)	21.5 (14.4–26.8)	20.4 (15.2–24.1)	0.08
Chronic infection by *Pseudomonas aeruginosa* in past 12 months, *n* (%)	13 (41.9)	7 (36.8)	6 (50)	1
**Comorbidities**				
ABPA, *n* (%)	4 (12.9)	2 (10.5)	2 (16.7)	0.63
CF liver disease, *n* (%)	7 (22.6)	5 (26.3)	2 (16.7)	0.67
CF related diabetes, *n* (%)	19 (61.3)	8 (42.1)	11 (91.7)	0.07
Systemic arterial hypertension, *n* (%)	6 (19.4)	1 (5.2)	5 (41.7)	0.02
**Treatments prior SARS-Cov-2 infection**				
Inhaled corticosteroids, *n* (%)	11 (35.5)	11 (57.8)	-	NA ^4^
Oral corticosteroids, *n* (%)	16 (51.6)	4 (21.1)	12 (100)	**<0.0001**
NSAIDs, *n* (%)	1 (3.2)	0 (0.0)	1 (8.3)	0.39
ACE inhibitors, *n* (%)	5 (16.1)	1 (5.2)	4 (33.3)	0.06
CFTR Modulators ^3^, *n* (%)	7 (22.6)	7 (36.8)	-	NA ^5^
Azithromycin, *n* (%)	18 (58.1)	11 (57.9)	7 (58.3)	1

^1^ GLI equations (14); ^2^ WHO2007 reference; ^3^ 2 ivacaftor; 4 lumacaftor-ivacafor; 1 tezacaftor-ivacaftor; ^4^ NA: not appropriate as post-lung transplant patients all receive oral corticosteroids, and thus no inhaled corticortseroids; ^5^ NA: not appropriate as CFTR modulators are not indicated in post-lung transplant patients. Sub-section headings are indicated in bold. Abbreviations: CF: cystic fibrosis; CFTR: cystic fibrosis transmembrane conductance regulator; ppFEV_1:_ percent-predicted forced expiratory volume in 1 s; BMI: body mass index; ABPA: allergic bronchopulmonary aspergillosis; NSAIDs: nonsteroidal anti-inflammatory drugs; ACE: angiotensin-converting enzyme.

**Table 2:** Evolution of the 31 patients with cystic fibrosis after SARS-CoV-2 infection.

	All Patients	Non-Transplanted Patients	Post-Lung Transplant Patients	Non-Transplanted vs. Post-Lung Transplant Patients
	*n* = 31	*n* = 19	*n* = 12	*p*-Value
**Patients’ management**				**0.008**
Ambulatory care, *n* (%)	12 (38.7)	11 (57.8)	1 (8.3)	
Hospitalization, *n* (%)	19 (61.3)	8 (42.1)	11 (91.7)	
**Hospitalization setting**				**0.6**
Medical ward (non-ICU), *n* (%)	15 (79)	7 (36.4)	8 (66.7)	
ICU, *n* (%)	4 (21)	1 (5.2)	3 (25)	
**Hospitalization duration** (days), median (range)	10 (2–41)	8 (2–19)	18 (3–41)	**0.06**
Patients discharge, *n* (%)				0.17
<10 days, *n*	9 (29)	5 (26.3)	4 (33.3)	
10–19 days, *n*	5 (16.1)	3 (15.7)	2 (16.7)	
20–29 days, *n*	4 (12.9)	0	4 (33.3)	
>30 days, *n*	1 (3.2)	0	1 (8.3)	
**Respiratory support**				
Additional oxygen therapy	7 (22.6)	1 (5.2)	6 (50)	0.006
Non-invasive ventilation (BIPAP, CPAP)	0	0	0	--
High flow nasal canula oxygen therapy	2 (6.5)	1 (5.2)	1 (8.3)	1
Invasive Ventilation	1 (3.2)	0	1 (8.3)	0.38
ECMO	0	0	0	--
**Additional treatments**				
Antiviral	0	0	0	--
Additional IV antibiotics	10 (32.3)	5 (26.3)	5 (41.7)	0.45
Additional oral antibiotics	11 (35.5)	7 (36.4)	4 (33.3)	1
Additional Azithromycin	5 (16.1)	4 (21.5)	1 (8.3)	0.62
Antifungal	1 (3.2)	0	1 (8.3)	0.39
Additional systemic corticosteroids	4 (12.9)	0	4 (33.3)	0.016
Hydroxychloroquine	2 (6.5)	0	2 (16.7)	0.14
Sarilumab	1 (3.2)	0	1 (8.3)	0.39
**Respiratory complications**	10 (32.3)	7 (36.4)	3 (25)	**0.69**
CF respiratory exacerbation	4 (12.9)	1 (5.2)	2 (16.7)	0.54
Bacterial pneumonia	2 (6.5)	1 (5.2)	1 (8.3)	1
ARDS	2 (6.5)	0	2 (16.7)	0.14
Hemoptysis	1 (3.2)	1 (5.2)	0	1
**Overall evolution**				
Recovered without short-term sequelae	31 (100)	19 (100)	12 (100)	--
Died	0	0	0	--

Sub-section headings are indicated in bold. Abbreviations: ICU: Intensive Care Unit; BIPAP: bilevel positive airways pressure; CPAP: continuous positive airway pressure; ECMO: extracorporeal membrane oxygenation; IV: intravenous.

## Supplementary Materials

The following are available online at https://www.mdpi.com/2077-0383/9/11/3624/s1, Figure S1: Observed and expected number of COVID-19 cases in French patients with cystic fibrosis according to age class, Table S1: COVID-19 cumulated incidence in the French general population, number and age (expected and observed) of COVID-19 in French cystic fibrosis patients, Table S2: Symptoms of the 11 CF patients for whom the diagnostic of SARS-Cov-2 infection was assessed by a positive serology, Table S3: Comparison of the clinical characteristics of the 31 CF patients at SARS-CoV-2 infection onset to the overall French CF population from the national registry, Table S4: Baseline clinical characteristics of the 4 patients who required ICU care, Table S5: Evolution of the 4 patients who required ICU care.
