# Supplementary material for: First Wave of COVID-19 in French Patients with Cystic Fibrosis
Source: J Clin Med. 2020 Nov 10;9(11):3624. doi: 10.3390/jcm9113624 (PMC7697588; doi:10.3390/jcm9113624)
Supplement: Supplementary file 1 [file jcm-09-03624-s001.pdf]

Supplementary material

First Wave of COVID-19 in French Patients with Cystic Fibrosis

Harriet Corvol [1,2,*], Sandra de Miranda [3], Lydie Lemonnier [4], Astrid Kemgang [2], Martine Reynaud Gaubert [5,6], Raphael Chiron [7], Marie-Laure Dalphin [8], Isabelle Durieu [9], Jean-Christophe Dubus [10], Véronique Houdouin [11], Anne Prevotat [12], Sophie Ramel [13], Marine Revillion [14], Laurence Weiss [15], Loic Guillot [2], Pierre-Yves Boelle [16] and Pierre-Régis Burgel [17,18] on behalf the French Cystic Fibrosis Reference Network study group

Supplementay material

Expected Number and Age Distribution of COVID-19 in French Patients with Cystic Fibrosis

We obtained the cumulated number of hospitalized COVID-19 cases in the French general population up to 30 June 2020. These data are open source, available from data.gouv.fr (https://www.data.gouv.fr/fr/datasets/r/41b9bd2a-b5b6-4271-8878-e45a8902ef00). Then, we obtained the probability of hospitalization upon SARS-Cov-2 infection by age from Table S1 in Salje et al. [21]. The numbers of people in each age class in France were obtained from census data (https://www.insee.fr/fr/statistiques/1892086?sommaire=1912926). From these data, we computed the extrapolated percentage of infections by age class up to 30 June 2020 in the general French population using proportionality rules. Finally, we computed the expected number of infections that would have been expected in the French CF population had the risk of infection been the same as in the general population. We used the number of CF patients from the national registry as the denominator to compute the expected number of SARS-Cov-2 CF cases [1]. The overall reduction in infection risk with respect to the general population was computed by comparing the expected number of cases with the observed number of cases (Table S1). We did not attempt to compute intervals as the sampling properties of most quantities are unknown.

Table S1. COVID-19 cumulated incidence in the French general population, number and age (expected and observed) of COVID-19 in French cystic fibrosis patients.

**COVID-19 in the French general population**									
**Age-class (years)**	**≤10**	**11–20**	**21–29**	**30–39**	**40–49**	**50–59**	**60–69**	**70–79**	**≥80**
Number of hospitalizations, n	726	536	2263	4350	7042	12,350	16,559	18,904	31,605
Risk of hospitalization upon infection (%)	(0.1%)	(0.1%)	0.5%)	(1.1%)	(1.4%)	(2.9%)	(5.8%)	(9.3%)	(26.2%)
Cumulated incidence of SARS-CoV-2 infection, %	9.4%	6.4%	6.1%	4.8%	5.9%	4.8%	3.6%	3.6%	2.9%

**COVID-19 in French patients with cystic fibrosis**					
**Age-class (years)**	**≤10**	**11–20**	**21–29**	**30–49**	**≥50**
Expected number of cases, n	150	120	100	90	10
Expected age distribution, %	32%	26%	21%	19%	2%
Observed number of cases, n	2	4	9	14	2
Observed age distribution	6%	13%	29%	45%	6%
**Overall risk reduction in COVID-19**			**93%**		

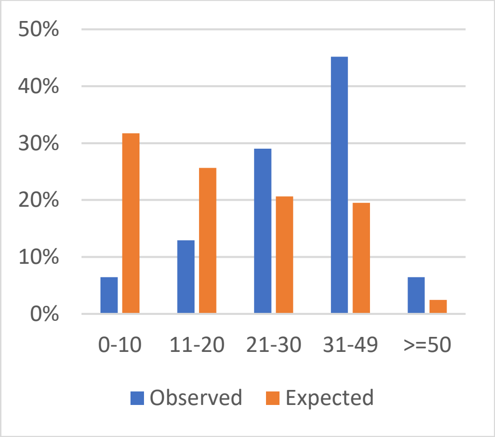

Figure S1. Observed and expected number of COVID-19 cases in French patients with cystic fibrosis according to age class.

Supplementay tables

Table S2. Symptoms of the 11 CF patients for whom the diagnostic of SARS-Cov-2 infection was assessed by a positive serology.

Patients Diagnosed by Positive Serology	n = 11
**Asymptomatic patients**, n (%)	3 (27.3)
**Symptomatic patients**, n (%)	8 (72.7)
Fever	4 (50)
Muscle ache/joint pain	2 (25)
Fatigue	5 (62.5)
Headache	3 (37.5)
Loss of taste	1 (12.5)
Increased cough	7 (87.5)
Increased dyspnea	1 (12.5)
Increased sputum production	3 (37.5)
Hemoptysis	1 (12.5)

Sub-section headings are indicated in bold.

Table S3. Comparison of the clinical characteristics of the 31 CF patients at SARS-CoV-2 infection onset to the overall French CF population from the national registry.

	CF Patients Infected by SARS-Cov-2 n = 31	Patients from the French CF Registry n = 6913	*p*-Value
Male, n (%)	48.4%	52.1%	0.82
Age (years), median (range)	31 (9-60)	20.3 (0.1, 85.1)	<0.0001
Exocrine pancreatic insufficiency, %	90.3%	80.3%	0.24
***CFTR* mutations**			0.48
F508del homozygotes, %	32.3%	41.4%	
F508del heterozygotes, %	51.6%	41.2%	
Other	16.1%	17.3%	
Influenza vaccine in the past 12 months, %	70.9%	NA	
$ppFEV_1$ (1), median	79%	83.3%	0.28
BMI (2), median	20.8%	21.2%	0.98

Chronic infection by *Pseudomonas aeruginosa* in past 12 months, %	41.9%	20.5%	0.007
**Comorbidities**			
ABPA, %	12.9%	9.5%	1.0
CF liver disease, %	22.6%	22.5%	1.0
CF related diabetes, %	61.3%	19.2%	<0.0001
Systemic arterial hypertension, %	19.4%	NA (3)	--
**Treatments prior SARS-Cov-2 infection**			
Inhaled corticosteroids, %	35.5%	36.9%	1.0
Oral corticosteroids, %	51.6%	13.7%	<0.0001
NSAIDs, %	3.2%	NA (3)	--
ACE inhibitors %	16.1%	NA (3)	--
CFTR Modulators, %	22.6%	17.9%	0.66
Azithromycin, %	58.1%	34%	0.009

(1) GLI equations (14); (2) WHO2007 reference; (3) NA: not available in the French CF registry [1]. Subsection headings are indicated in bold. Abbreviations: CF: cystic fibrosis; CFTR: cystic fibrosis transmembrane conductance regulator; $ppFEV_1$: percent-predicted forced expiratory volume in 1 s; BMI: body mass index; ABPA: allergic bronchopulmonary aspergillosis; NSAIDs: nonsteroidal anti-inflammatory drugs; ACE: angiotensin-converting enzyme.

Table S4. Baseline clinical characteristics of the 4 patients who required ICU care.

	**All Patients n = 4**	**Non-Transplanted Patients n = 1**	**Post-Lung Transplant Patients n = 3**
Male, n (%)	4 (100)	1	3 (100)
Age (years), median (range)	45 (9–48)	9	46 (43–48)
Exocrine pancreatic insufficiency, n (%)	4 (100)	1	3 (100)
CFTR mutations			
F508del homozygotes, n (%)	1 (25)	0	1 (33.3)
F508del heterozygotes, n (%)	2 (50)	1	1 (33.3)
Other	1 (25)	0	1 (33.3)
Post-lung transplant	3 (75)	0	3 (100)
Influenza vaccine in the past 12 months, n (%)	4 (100)	1	3 (100)
$ppFEV_1$ *, median (range)	75 (34–91)	75	58 (34–91)
BMI **, median (range)	17.78 (16.5–20.3)	16.6	19.4 (17.8–20.4)
Chronic infection by *Pseudomonas aeruginosa* in past 12 months, n (%)	1 (25)	1	1 (33.3)
Comorbidities			
ABPA, n (%)	0	0	0
CF liver disease, n (%)	0	0	0
CF related diabetes, n (%)	1 (25)	0	2 (66.7)
Systemic arterial hypertension, n (%)	2 (50)	0	2 (66.7)
Treatments prior SARS-Cov-2 infection			
Inhaled corticosteroids, n (%)	1 (25)	1	0
Oral corticosteroids, n (%)	3 (75)	0	3 (100)
NSAIDs, n (%)	1 (25)	0	1 (33.3)
Immunosuppressive drugs, n (%)	3 (75)	0	3 (100)
ACE inhibitors, n (%)	2 (50)	0	2 (66.7)
CFTR Modulators, n (%)	0	0	0
Azithromycin, n (%)	3 (75)	0	3 (100)

* GLI equations (14); ** WHO2007 reference. Abbreviations: CF: cystic fibrosis; CFTR: cystic fibrosis transmembrane conductance regulator; $ppFEV_1$: percent-predicted forced expiratory volume in 1 s; BMI: body mass index; ABPA: allergic bronchopulmonary aspergillosis; NSAIDs: nonsteroidal anti-inflammatory drugs; ACE: angiotensin-converting enzyme.

Table S5. Evolution of the 4 patients who required ICU care.

	All Patients n = 4	Non-Transplanted Patients n = 1	Post-Lung Transplant Patients n = 3
**Hospitalization duration (days), median (range)**	**27 (16-29)**	**16**	**28 (26-29)**
**Patients discharge, n (%)**			
<10 days, n	0	0	0
10–19 days, n	1 (25)	1	0
20–29 days, n	3 (75)	0	3 (100)
>30 days, n	0	0	0
**Respiratory support**			
Additional oxygen therapy	3 (75)	0	3 (100)
Non-invasive ventilation (BIPAP, CPAP)	0	0	0
High flow nasal canula oxygen therapy	1 (25)	0	1 (33.3)
Invasive ventilation	1 (25)	0	1 (33.3)
ECMO	0	0	0
**Additional treatments**			
Antiviral	0	0	0
Additional IV antibiotics	3 (75)	1	2 (66.7)
Additional oral antibiotics	1 (25)	1	0
Additional Azithromycin	1 (25)	1	0
Antifungal	0	0	0
Additional systemic corticosteroids	2 (50)	0	2 (66.7)
Hydroxychloroquine	0	0	0
Sarilumab	1 (25)	0	1 (33.3)
**Complications**	3 (75)	1	2 (66.7)
CF respiratory exacerbation	1 (25)	0	1 (33.3)
Bacterial pneumonia	1 (25)	1	0
ARDS	2 (50)	0	2 (66.7)
Encephalopathy	1 (25)	0	1 (33.3)
Renal failure	1 (25)	0	1 (33.3)
**Overall evolution**			
Recovered without sequelae	4 (100)	1 (100)	3 (100)
Died	0	0	0

Sub-section headings are indicated in bold. *Abbreviations:* BIPAP: bilevel positive airways pressure; CPAP: continuous positive airway pressure; ECMO: extracorporeal membrane oxygenation; IV: intravenous, ARDS: acute respiratory distress syndrome.
